# Eating Behaviors in Relation to Child Weight Status and Maternal Education

**DOI:** 10.3390/children8010032

**Published:** 2021-01-07

**Authors:** Priscilla Ayine, Vaithinathan Selvaraju, Chandra M. K. Venkatapoorna, Yida Bao, Philippe Gaillard, Thangiah Geetha

**Affiliations:** 1 Department of Nutrition, Dietetics, and Hospitality Management, Auburn University, Auburn, AL 36849, USA; pza0022@tigermail.auburn.edu (P.A.); vzs0041@auburn.edu (V.S.); iamchandru@gmail.com (C.M.K.V.); 2 Department of Mathematics and Statistics, Auburn University, Auburn, AL 36849, USA; yzb0010@auburn.edu (Y.B.); prg0007@auburn.edu (P.G.); 3 Boshell Metabolic Diseases and Diabetes Program, Auburn University, Auburn, AL 36849, USA

**Keywords:** child eating behavior, childhood obesity, maternal education, food avoidance, food approach

## Abstract

Background: The eating behavior of children is important to maintain a healthy weight. This current study explored the differences in children’s eating behaviors and their relation to weight status and maternal education level, using the child eating behavior questionnaire (CEBQ). Methods: The study recruited 169 participants aged between six and ten years. Multinomial logistic regression was conducted to examine the association between the CEBQ factors and children’s body weight status. The association between the CEBQ scores and maternal educational levels was examined using a one-way analysis of variance (ANOVA). Results: The multinomial logistic regression findings indicate that children in the obese group exhibited a significant increase in food responsiveness, enjoyment of food, emotional overeating, and a decrease in satiety responsiveness compared to normal weight children. The one-way ANOVA showed a significant difference in subscales under the food approach (food responsiveness, desire to drink, emotional overeating) and food avoidance (satiety responsiveness) based upon the child’s weight status. The three subscales under the food approach category were significantly dependent upon the maternal education but did not have a significant association with food avoidance. Conclusions: The results suggest that the increase in food responsiveness and emotional overeating in obese children is influenced by maternal education.

## 1. Introduction

Despite researchers’ significant efforts to combat obesity, it remains a critical health concern worldwide as its prevalence keeps rising steadily [1]. Obesity is a metabolic disorder due to the accumulation of body fat, and it is usually measured using the World Health Organization (WHO) classification of body mass index (BMI). BMI is defined as the ratio of body weight in kilograms (kg) to height in meter squared (m^2^). For instance, an adult with a BMI of <18.5 kg/m^2^ is classified as underweight, 18.5–24.9 kg/m^2^ is classified as normal weight, and >30 kg/m^2^ is considered obese [2]. Overweight and obesity are considered risk factors for various chronic diseases such as type 2 diabetes, high blood pressure, heart diseases, stroke, and some forms of cancer [3,4,5,6,7]. Obesity is caused by genetic, behavioral, environmental (for example geography, food availability, and transportation), and socioeconomic factors [8]. 

Around 12.7 million children and adolescents are classified as obese in the United States [9]. Childhood obesity is often persistent into adulthood [10]. Childhood obesity is often defined as BMI ≥95th percentile [11], and one in five children are considered overweight or obese globally [12], which translates to 41 million infants and young children as reported by a WHO 2016 study [2]. It is reported that a child is 10 to 12 times more likely to be obese when they have two parents with obesity compared to having parents with a healthy weight [13,14]. Not only is parental obesity linked to obesity in their children, but it has also been shown to be implicated in the etiology of eating disorders (Eds), including bulimia nervosa [15], binge-eating disorder [16], and anorexia nervosa [17]. Furthermore, studies indicate that children of mothers with overweight and obesity demonstrate higher emotional eating levels than children of healthy weight mothers [18]. Mothers especially assist their children in learning and developing both eating behaviors and food preferences [19]; it has been reported that children varied their food choices depending on whether they were being observed by their parents or not [20]. In several studies, examining the relationship between maternal education and diet in infants and children revealed that higher educational status was related to the extended duration of breastfeeding, enhanced physical growth, higher intakes of micronutrients, fruits, and vegetables, and lower intake of sugary drinks [21,22,23,24,25]. 

Another study revealed maternal education as a strong determinant for better nutritional intake in primary school children aged 7–10 years [25]. Additionally, it has been suggested that a child’s diet and food preferences are typically influenced by food environments, including their parents’ eating behaviors [23,26]. This influence is strongest in early childhood, where parents serve as the gatekeepers and role models around food [27,28]. It tackles disordered eating, which is established to be a cause of childhood obesity. It is essential to understand and positively influence the modifiable determinants of healthy eating behaviors in early life [26,29]. 

Additionally, maternal education is positively associated with healthier diets not only for mothers themselves [23,30,31,32], but in their young children under 2 years old [32,33,34] and older as well [23,31,35,36]. Moreover, low maternal education was found to yield a significant risk of early childhood adiposity [37]. Despite research showing the importance of healthy eating behaviors and the influence of maternal education on childhood weight gain, a great number of children in the United States and other countries has been found to keep poor dietary habits into adulthood [38]. The child eating behavior questionnaire (CEBQ) is a thoroughly compiled parent-report psychometric measure of an array of children’s appetitive characteristics to test this theory in samples of children [39]. The CEBQ is used to understand the insight into parents’ perceptions of their children’s eating behaviors [40]. A previous study using the CEBQ examined the association between the scores of the CEBQ and BMI in a sample of Portuguese children. They found that after controlling for gender, age, and socioeconomic status, all the subscales of CEBQ were significantly associated with BMI z-scores [41]. Jansen et al. [42] used one factor of the CEBQ to assess directionality in the relation between fussy eating parents’ pressures on children to eat. The CEBQ is widely used to examine differences in children’s appetite in relation to their body weight.

However, not much has been done to examine differences in children’s eating behaviors in relation to child weight status and maternal level of education. Additionally, given that childhood obesity increases the chances of becoming obese in one’s adulthood, the urgent need to understand eating behaviors in children which has been shown to be influenced by maternal education is emphasized [38]. Therefore, in this study, we aimed to examine (1) the association between the CEBQ and child weight status and (2) the differences in child appetite characteristics in relation to children’s body weight and maternal education level.

## 2. Materials and Methods

### 2.1. Participants

Participants (*n* = 169) aged six to ten years (mean 8.42 ± 0.10) were recruited from the Lee and Macon county area in Alabama. The same participants were used in our previously published articles [43,44]. Therefore, the general characteristics of the study population are presented in those articles. Participants were recruited by posting flyers at after-school programs, childcare, Facebook, as well as through a participant’s referral. Exclusion criteria included children with significant health disorders such as diabetes, cardiovascular disease, or diagnosed sleep disorder based on a prior phone survey with parents. The parents brought their children to Auburn University to participate in the study. The appropriate sample size was estimated using G*Power 3.1.9.4 [43]. Written consent was obtained from both parents and children. The Auburn University Institutional Review Board approved the study.

### 2.2. Anthropometric Measures

Anthropometric measurements such as the body weight and height of the participants were measured according to the WHO recommendations. Body weight was recorded with light clothing using a Tanita digital scale (WB-800H plus), and height was measured using a stadiometer attached with a scale. BMI was calculated using the recorded height and weight as per the Centers for Diseases Control and Prevention (CDC) growth chart. The participants were grouped into normal weight (NW), overweight (OW), and obese (OB) based on the BMI percentile charts for age and sex from the CDC [45]. The details of the measurement are given in our previous studies [43,44]. 

### 2.3. Psychometric Measures

The CEBQ is a 35-item instrument initially designed and validated to measure a range of children’s eating behaviors [39]. The instrument is shown to have good internal consistency, test–retest reliability, and stability over time [39,46]. It has been shown to be related to food intake in the behavioral test [47]. The questionnaire includes four subscales that measure food approach, which consist of food responsiveness (FR), enjoyment of food (EF), emotional overeating (EOE), and desire to drink (DD). It also includes four other subscales that measure food-avoidant-type responses, which include satiety responsiveness (SR), slowness in eating (SE), emotional undereating (EUE), and food fussiness (FF). In a validation study by Wardle et al. [39], SR and SE were found to load onto the same factor, so they have been combined to form a single scale. Parents on behalf of the participants completed the CEBQ. They were provided with options to provide response between “never” and “always” on a 0–4 Likert-type scale. Scale scores were calculated if at least more 75% of the items were completed [42], and subscales of each factor were averaged and used for analysis.

### 2.4. Statistical Analysis

All data were analyzed using Statistical Package for the Social Science (SPSS), version 25 (IBM Corp., Armonk, NY, USA). The internal reliability (Cronbach’s alpha) analysis was performed for the different scales of the CEBQ instrument. Pearson’s correlation was commuted to estimate the relationships between the mean items scale scores on each of the seven factors of the CEBQ questionnaire, and interpretation of the correlation coefficients was made according to Cohen’s descriptive guidelines [48]. Correlations between 0.1 and 0.3 were interpreted as small, correlations between 0.3 and 0.5 considered medium, and correlations between 0.5 and 1.0 large. The multinomial logistic regression was conducted to examine associations between scores of CEBQ subscales with children’s body weight status. Children’s weight category was the dependent variable, and NW was used as a reference point to the OW and OB groups. The difference in children’s weight status and maternal educational levels between CEBQ scores was ascertained using one-way ANOVA. The *p*-values generated were against normal weight category. 

## 3. Results

In this study, 64.5% of the participants were NW, 18.9% were OW, and 16.6% were OB. The ages of participants ranged from 6 to 10 years with a mean age of the study sample being 8.42 years. There were no significant differences observed in age, gender, or maternal education. The characteristics of the participants and maternal education are given in our previously published manuscripts [43,44]. Internal reliability coefficients (Cronbach’s alpha) for the different scales of the CEBQ are presented in Table 1 below. The Cronbach’s alpha coefficients of the CEBQ subscales ranged from 0.784 to 0.915 and were all within acceptable range [49], which indicates that the CEBQ results have relatively high internal consistency.

Pearson bivariate correlations between CEBQ subscales are presented in Table 2. The correlations between the subscales indicate that food-avoidant subscales (satiety responsiveness) were negatively correlated to food approach subscales (food responsiveness (*r* = −0.477, *p* < 0.001) and enjoyment of food (*r* = −0.495, *p* < 0.001)) and positively correlated to food-avoidant subscale emotional undereating (*r* = 0.267, *p* < 0.001). Another food avoidance subscale, food fussiness, was also negatively correlated to food approach subscales food responsiveness (*r* = −0.118) and enjoyment of food (*r* = −0.513, *p* < 0.001). These were positively correlated to food-avoidant subscale emotional undereating (*r* = 0.273, *p* < 0.001). The food approach subscale food responsiveness was positively correlated to other food approach subscales such as enjoyment of food (*r* = 0.418, *p* < 0.001), desire to drink (r = 0.294, *p* < 0.001), and emotional overeating (*r* = 0.53, *p* < 0.001). Another positive correlation between food approach subscales was desire to drink and emotional overeating (*r* = 0.279 *p* < 0.001). In addition, one food-avoidant subscale, emotional undereating, and food approach subscale emotional overeating were positively correlated (*r* = 0.281, *p* < 0.001).

Multinomial logistic regression was used to examine the association between subscale scores of CEBQ and children’s body weight. The regression model was adjusted for confounding variables such as age, gender, and maternal education. A significant negative association was found with one food approach subscale (desire to drink) in the overweight category (β = −0.511, *p* = 0.01) and one food-avoidant subscale (satiety responsiveness) in the obese category (β = −1.18, *p* = 0.001). A positive association was found in the obese group with three food approach subscales, i.e., food responsiveness (β = 0.865, *p* = 0.001), enjoyment of food (β = 0.633, *p* = 0.037), and emotional overeating (β = 0.568, *p* = 0.032) in the obese group compared to normal weight (Table 3).

**Figure 1:**
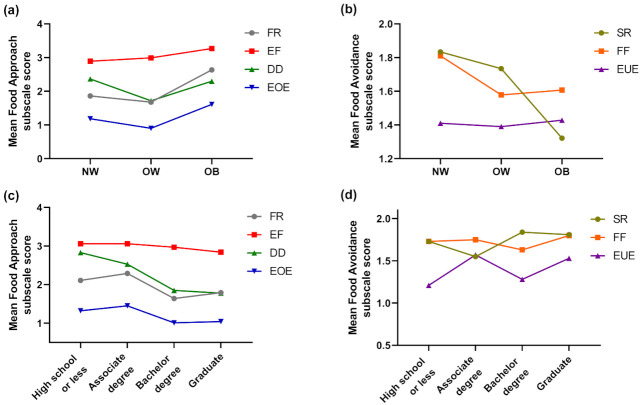
Mean scores of the subscales of food approach and food avoidance of the CEBQ based on (**a**,**b**) weight and (**c**,**d**) maternal education category.

Table 4 shows the mean scores of the CEBQ subscales based upon the children’s weight status and maternal education. Results obtained from the one-way ANOVA revealed significant differences in three subscales, i.e., food responsiveness, desire to drink, and emotional overeating under food approach, which measure a child’s interest in food with BMI. However, food responsiveness (*p* = 0.001) and emotional overeating (*p* = 0.003) were significantly increased in the OB group compared to NW and OW. Only one subscale, satiety responsiveness, under food avoidance, which measures children’s lack of interest in food, was decreased significantly (*p* = 0.002) in the obese group compared to NW and OW (Figure 1a,b).

In relation to maternal education in children’s eating behaviors, the results reveal significant differences only in the food approach subscales, i.e., food responsiveness (*p* = 0.008), desire to drink (*p* = 0.001), and emotional overeating (*p* = 0.037). The mean scores showed that the children of mothers with a Bachelor’s degree or graduate-level education had significantly decreased food responsiveness (*p* = 0.008), desire to drink (*p* = 0.0001), and emotional overeating (*p* = 0.037) compared to mothers with a lower education level (Figure 1c,d).

## 4. Discussion

This study investigated whether children’s eating behaviors are different based on their weight status and maternal educational level. The findings revealed that obese children exhibited a higher interest in food due to increased scores of food responsiveness, enjoyment of food, and emotional overeating. Children with obesity were revealed to score relatively lower in satiety responsiveness, which measures children’s lack of interest in food compared to that of children of normal weight. The previous results theoretically support these findings [41,50,51]. 

The relationship between these children’s eating factors and maternal education was also evaluated. Findings in the mean scores for maternal education revealed that children of mothers with a Bachelor’s degree or graduate level education showed significantly decreased food responsiveness, desire to drink, and emotional overeating compared to those of mothers with a lower education level. The food approach subscales were only dependent upon maternal education and not food avoidance. This is consistent with studies that have indicated that greater educational achievement among mothers may reduce their children’s susceptibility to the cumulative nature of obesogenic factors [52,53,54,55].

Multinomial logistic regression testing the mean-scale scores revealed that children with obesity exhibited positive responsiveness, showing greater pleasure in eating, and emotionally overeating. The positive association of these food approach scales (food responsiveness, enjoyment of food, emotional overeating) with the BMI z-score is similar to results reported by others that demonstrate children with a higher BMI z-score tend to be greatly receptive to environmental food cues [39,41,50,51,56]. The only food approach subscale that revealed a negative association with the BMI z-score was desire to drink.

The present study has limitations; one of several is the location of the study. The study was carried out on the Auburn University campus. Therefore, participants were recruited in and around Auburn, Opelika, Beauregard, and Tuskegee in Alabama. In a college town, it is worth mentioning that parents may well be more interested in nutrition education compared to parents in non-college rural areas. Therefore, these findings may not be generalizable. Another limitation is the self-reporting nature of the CEBQ questionnaire, indicating that parents subjectively reported the children’s eating behaviors. Moreover, this was not a longitudinal or experimental study; thus, inferences concerning causality should not be made. These findings show that more studies are needed to investigate the role of eating behaviors in the etiology of obesity in early childhood by hypothesizing whether individual eating style is the reason for weight gain or vice versa.

To the best of our knowledge, this is one of the few studies exploring the differences in individual eating styles in children that also includes maternal educational levels. Our findings indicate that there is an individual feeding difference between children of different weight status, and maternal educational level. However, further investigation is needed to determine the direction of the effect. Our findings may help in gaining insights into understanding the many pathways contributing to the etiology of early child weight gain, thereby designing appropriate nutrition interventions that will promote healthy feeding behaviors in children.

## 5. Conclusions

Our findings suggest that the increase in food responsiveness and emotional overeating in obese children are influenced by maternal education. Given that there is an association between maternal education and eating behavior in children, our results suggest that it is essential to compensate for the level of maternal education. 

## Figures and Tables

**Table 1:** Factor structure and internal reliability of the child eating behavior questionnaire.

CEBQ Subscales	Mean ± SE	Cronbach
Food Responsiveness (FR)	1.96 ± 0.07	0.815
Enjoyment of Food (EF)	2.97 ± 0.06	0.798
Desire to Drink (DD)	2.23 ± 0.09	0.915
Emotional Overeating (EOE)	1.20 ± 0.06	0.872
Satiety Responsiveness (SR)	1.73 ± 0.05	0.784
Food Fussiness (FF)	1.73 ± 0.08	0.906
Emotional Undereating (EUE)	1.41 ± 0.07	0.796

SE—Standard error.

**Table 2:** Pearson’s correlations between the subscales of child eating behavior questionnaire.

CEBQ Subscales	FR	EF	DD	EOE	SR	FF	EUE
1. Food responsiveness	1.000						
2. Enjoyment of food	0.418 *	1.000					
3. Desire to drink	0.294 *	0.094	1.000				
4. Emotional overeating	0.530 *	0.144	0.279 *	1.000			
5. Satiety responsiveness	−0.477 *	−0.495 *	−0.045	−0.130	1.000		
6. Food fussiness	−0.118	−0.513 *	0.078	0.009	0.378 *	1.000	
7. Emotional undereating	0.007	−0.251 *	0.136	0.281 *	0.267 *	0.273 *	1.000

The statistical significance in the table is shown as * *p* < 0.001 level.

**Table 3:** Multinomial regression analysis for the overweight and obese groups (reference category: normal weight (NW)) in the child eating behavior questionnaire (CEBQ) subscale (adjusted for age, gender, and maternal education).

	OW	*p* Value	OB	*p* Value
**Food approach**				
Food responsiveness (FR)	−0.186 (0.24)	0.44	0.865 (0.26)	**0.001**
Enjoyment of food (EF)	0.172 (0.26)	0.51	0.633 (0.30)	**0.037**
Desire to drink (DD)	−0.511 (0.20)	**0.01**	−0.173 (0.20)	0.39
Emotional overeating (EOE)	−0.528 (0.29)	0.07	0.568 (0.26)	**0.032**
**Food avoidance**				
Satiety responsiveness (SR)	−0.303 (0.32)	0.34	−1.180 (0.37)	**0.001**
Food fussiness (FF)	−0.219 (0.20)	0.27	−0.134 (0.21)	0.53
Emotional undereating (EUE)	−0.037 (0.22)	0.87	0.06 (0.24)	0.80

Normal weight (NW), overweight (OW), obese (OB). Values are represented as β coefficient (SE). *p* < 0.05 considered significant and represented in bold.

**Table 4:** One-way ANOVA of factors based on body weight and maternal education.

	Food Approach				Food Avoidance		
	FR	EF	DD	EOE	SR	FF	EUE
**Body Mass Index**							
NW (*n* = 109)	1.86(0.08)	2.89(0.80)	2.36(0.11)	1.19(0.08)	1.83(0.07)	1.81(0.10)	1.41(0.09)
OW (*n* = 32)	1.68(0.16)	2.99(0.12)	1.72(0.18)	0.90(0.13)	1.73(0.10)	1.58(0.18)	1.39(0.17)
OB (*n* = 28)	2.63(0.20)	3.27(0.14)	2.30(0.22)	1.61(0.18)	1.32(0.13)	1.61(0.21)	1.43(0.17)
*p*-value	**0.001**	0.086	**0.021**	**0.003**	**0.002**	0.435	0.988
**Maternal Education**							
High school or less (*n* = 40)	2.11(0.16)	3.06(0.13)	2.83(0.17)	1.32(0.17)	1.73(0.09)	1.73(0.14)	1.21(0.13)
Associate degree (*n* = 42)	2.29(0.14)	3.06(0.13)	2.53(0.17)	1.45(0.13)	1.55(0.12)	1.75(0.18)	1.57(0.14)
Bachelor degree (*n* = 37)	1.64(0.14)	2.97(0.12)	1.85(0.18)	1.01(0.11)	1.84(0.11)	1.63(0.19)	1.28(0.17)
Graduate (*n* = 50)	1.79(0.13)	2.84(0.12)	1.78(0.15)	1.04(0.09)	1.81(0.10)	1.80(0.14)	1.53(0.13)
*p*-value	**0.008**	0.499	**0.001**	**0.037**	0.217	0.909	0.220

Results are expressed as mean (SE). *p* < 0.05 considered significant and represented in bold. Food responsiveness (FR); enjoyment of food (EF); desire to drink (DD); emotional overeating (EOE); satiety responsiveness (SR); food fussiness (FF); emotional undereating (EUE).

## Data Availability

The data presented in this study is available on request from the corresponding author.
